# Mr. Vandier, on the Kilburn Waters

**Published:** 1801-12

**Authors:** F. N. Vandier

**Affiliations:** Great Mary le Bone Street


### To the Editors of the Medical and Phyſical Journal.

GENTLEMEN,

WHEN I wrote the obſervations on the Analyſis of the Kilburn Waters, which have been inſerted in the Number of your Journal for Auguſt, I was fully convinced of their accuracy, and I was actuated ſolely by a deſire of pointing out what I thought erroneous, and to caution your younger readers againſt the concluſions drawn by the author of the analyſis. That gentleman having, in anſwer to my animadverſions, quoted authorities in oppoſition to that of M. Fourcroy, I find myſelf obliged to trouble you again with ſome further remarks, in order that the candid and impartial public may decide upon a queſtion more important perhaps than it appears at firſt ſight. I truſt that my reaſons will be found ſufficient to remove all doubt; I therefore ſubmit them to your readers, aſſuring you at the ſame time that it will be always my wiſh, "*Et refellere sine pertinaciâ, et refelli sine iracundiâ,*" truth being dearer to me than any opinion.

I had

I had objected to the term "*Strontian lime*," as being in chemical language a barbarifm; the author replies, that he will not apologize for it, having the authorities of Meffrs. Kirwan, Babington, and Aikin. Thefe are indeed very refpectable authorities, but I hope I am not too prefumptuous in faying that no authority can avail againft philofophical rules; and that, if the new nomenclature is adopted at all, it fhould be employed as it exifts originally, or as corrected to the prefent time. Some would, perhaps, add, that Mr. Kirwan cannot be confidered as an authority in point of nomenclature, fince he has one of his own which the chemical world has not adopted; or fhall we fay with him, fuliginated, inftead of ammoniacal; Sylvian, inftead of muriate of potafh; Glauber and Epfom, inftead of fulphates of foda and of magnefia; mephito nitrous acid, inftead of nitric acid impregnated with nitrous gas, &c.?

But enough of thefe remarks about words, let us examine the moft important parts. It feems to me that the queftion to be decided is fimply this: "*Do muriate of lime and sulphates of soda and of magnesia exist together in the Kilburn Waters, and is it possible that they may co-exist?*" The author of the analyfis fays he has found them, and has brought authorities to fupport his affertion. You will recollect, gentlemen, that I related two fimple experiments to prove the contrary, and quoted "one of the moft fkilful and experienced hydro-analyfts of modern times," my illuftrious countryman, Profeffor Fourcroy.* I muft confefs that I have fince found no reafon to alter my opinion, however refpectable may be *some* of the authorities alluded to. For,

Firft, It may be obferved that Mr. Schmeiffer in particular cannot be quoted as proving any thing, it having been proved by the author that Mr. Schmeiffer was certainly wrong in one point of his analyfis, viz. in his afferting that the water contains fulphurated hydrogen gas.

Secondly, It may alfo be remarked that fome of the analyfes brought forwards were made at a time when analytical chemiftry was not fo far advanced as it is now.

Thirdly, If we appeal to Mr. Kirwan's book on the Analyfis of Waters, we find, that although that learned philofopher cautions us, by the expreffions quoted in the anfwer to my obfervations, againft concluding too haftily on the prefence or abfence of any falts in conjunction with others apparently incompatible, yet he every where tells us that muriate of lime cannot exift with alcaline fulphates, or with fulphate of magnefia.

* Thefe are Mr. Kirwan's expreffions, p. 159, *Analyfis of Waters*.

fia. "This falt (muriate of lime) is incompatible with any notable proportion of aerated alcalis or aerated magnefia, and *with all fulphats, except selenite*;" page 123. "If it (fulphate of magnefia) be accompanied with felenite and Glauber (fulphates of lime and of magnefia) neither then can any other earthy nitrate or muriate exift in the water, &c." page 216.

"The exiftence of fulphate of magnefia demonftrated in the water, although in very fmall quantity, proves that the muriatic acid cannot be united to lime, for the calcareous muriate cannot exift with fulphate of magnefia, without decompofing it and being in its turn decompofed, fo that thefe two falts are fuddenly changed into fulphate of lime and muriate of magnefia." Fourcroy, Analyfe des Eaux d'Enghien, p. 217.

M. Bertholet, in his "Recherches fur les Lois de l'Affinité," obferves alfo, that muriate of lime changes its bafis with all foluble fulphates.

Fourthly, I would afk how it is poffible to know that thefe falts, faid to be incompatible by all authors, did co-exift in the Kilburn or other waters? And, how far it is juftifiable to contradict the general law of chemical attraction on mere appearances? For their co-exiftence was concluded merely, I believe, on the obfervation that when they are in a very fmall quantity in a large proportion of water, there is no apparent decompofition. But it may be obferved, that chemical bodies do not act upon one another, unlefs they are brought into contact; and that, in the circumftances alluded to, the fmall quantities are loft, if I may fo fay, in an immenfe bulk of fluid, and cannot therefore fairly approach one another. This is well known to all Analyfts, who find themfelves frequently obliged to evaporate part of the folvent; but they do not hence conclude, that the bodies in folution do not act upon one another.

There are but two ways of afcertaining the prefence of thefe incompatible, yet (as is fuppofed) co-exifting falts. The *firft method* is by tefts or reagents. But Mr. Kirwan on the Analyfis of Waters, p. 159; Fourcroy, Analyfe des Eaux d'Enghien, p. 69; Gioanetti, Analyfe des Eaux de St. Vincents, page 31, and many other writers, agree that no certain conclufion can be drawn from reagents, and that they cannot give any information of the combinations. The *second method* is by evaporation; but here again Mr. Kirwan obferves very juftly, "That when, by clofe evaporation, they (the incompatible falts) are brought together in an inconfiderable fpace, they decompofe each other, &c." page 164.

Thefe reafonings were fully fufficient to perfuade me; but I went ftill further: I examined the Kilburn Waters, and I fhall give fuch of the experiments as relate to the prefent queftion, and and omit the others, ſince my intention is not to give the whole analyſis.

1. A gallon was evaporated till it was reduced to a half, and the carbonates were collected; they were found to weigh about 36 grains.

2. The liquor was then evaporated to dryneſs, and left expoſed to atmoſpheric air for twenty-four hours; no ſenſible deliqueſcence was obſerved, and the reſiduum, in that ſtate, weighed 501 grains.

3. That reſiduum was put with very dry alcohol, and filtered, twenty-four hours after it had been frequently ſhaken. The liquor was carefully diſtilled to dryneſs, and the reſiduum, diſſolved in diſtilled water, was perfectly tranſparent.

4. I then poured into it ſome ſulphuric acid, but not enough to rediſſolve the precipitate if there had been any; no precipitate was produced. In order to aſcertain whether the liquor was able to ſhow the lime, if there had been any, a ſmall drop of a ſolution of muriate of lime was added to it, and then a precipitate of ſulphate of lime was produced. This caution is always neceſſary before we can draw any concluſion.

5. The nitrate of ſilver demonſtrated the preſence of muriatic acid in the ſolution (Exp. 3.), and magneſia was precipitated by ſub-carbonate of pot-aſh.

Theſe experiments were repeated many times, and always with the ſame reſults.

As it might have been objected that I had not followed exactly the ſame method as the gentleman I had criticiſed, I evaporated another gallon to dryneſs, leaving the carbonates with the reſiduum, which, kept for five or ſix days on a ſand-bath, weighed 446 grains. It was then put with very dry alcohol, and after ſtanding forty-eight hours, it was filtered, diſtilled to dryneſs, and detached from the veſſel by diſtilled water. I then put it in a platina crucible, and poured on it ſome ſulphuric acid, which I expoſed for ſome time to a red heat; cold diſtilled water was added in a ſmall quantity, and no precipitate of any kind was perceived, but the ſolution was perfectly tranſparent, and was ſulphate of magneſia.

From the foregoing experiments, it ſeemed fair to conclude that muriate of lime and ſulphates of magneſia and of ſoda do not co-exiſt in the Kilburn Waters. I did not, however, reſt ſatisfied with theſe experiments, but thought it incumbent upon me to make ſuch others as would confirm the concluſions which I have drawn. A very natural method preſented itſelf; I mean the ſyntheſis or recompoſition of the waters. I am well aware that our inability to reproduce a compound which has been ana-analyſed, does not always prove that the analyſis is erroneous.

We

We cannot reproduce a ruby or a faphire; but it is otherwife with refpect to mineral waters. If the analyfis be correct, we may, by combining the different ingredients, form a compound exactly fimilar, at leaft as to the various falts which exift in it.

I therefore took a gallon of diftilled water, and boiled with it forty-two grains of fulphate of lime, till it was diffolved. When cold, the muriates and fulphates were added, then the carbonates, all in the proportions ftated in Mr. Blifs's Analyfis; carbonic acid gas was paffed through the liquor, till the carbonates were diffolved; the liquor was not inferior to that of the natural waters in point of limpidity, and the tafte appeared to be the fame. This artificial was then analyfed as the natural waters had been, and the 14,75 grains of muriate of lime, which had been put into it, could not be found by any method I could devife. It had therefore been decompofed, as I indeed expected; and this feems to me a fatisfactory proof that muriate of lime and fulphates of alcalies and magnefia cannot co-exift.

It muft be obferved, that the fulphuric acid ufed in thefe experiments was diftilled for that purpofe, and that no other can be depended on, as it is generally very impure, and contains often, befides other matters, fulphates of lead, of potafh, of alumina, &c. I had alfo occafion, in the courfe of my experiments, to obferve that the whole of the muriate of magnefia does not appear to be taken up by alcohol, when this laft is very dry, and when the falt is enveloped, as it were, by other falts, alfo very dry.

It appears alfo, that very dry alcohol takes up fome fulphate, for the muriate of barytes demonftrated a flight trace of fulphuric acid in one of the folutions which I examined, fo true is it that there is nothing abfolute.

I am, your's, &c.

F. N. VANDIER.

*Great Mary le Bone Street, Nov.* 11, 1801.

